# Research Progress on the Relationship between Obesity-Inflammation-Aromatase Axis and Male Infertility

**DOI:** 10.1155/2021/6612796

**Published:** 2021-02-08

**Authors:** Liu Yuxin, Lin Chen, Luo Xiaoxia, Luo Yue, Lai Junjie, Li Youzhu, Zhou Huiliang, Liu Qicai

**Affiliations:** ^1^School of Medical Technology and Engineering, Fujian Medical University, China; ^2^Center of Reproductive Medicine, The First Affiliated Hospital of Fujian Medical University, China; ^3^Center of Reproductive Medicine, The First Affiliated Hospital of Xiamen University, China

## Abstract

Aromatase is a key enzyme in the transformation of androgen into estrogen. Its high expression will destroy the hormonal balance in the male body, and the excessive transformation of androgen into estrogen in the body will further damage the spermatogenic function of the testis, affect the normal development of the sperm, and cause spermatogenic disturbance. Adipose tissue has a high expression of aromatase and shows high enzymatic activity and ability to convert estrogen. Adipose tissue is the most estrogen-producing nongonadal tissue in the body because of its large size, accounting for about 20% of the body mass in healthy adults. PPAR*γ* is recognized as the key adipose differentiation in the transcriptional regulation of the transcription factor. In the process of adipocyte differentiation, PPAR*γ* regulate the expression of aromatase. The increase of aromatase is associated with the inflammatory response in adipose tissue caused by obesity. After obesity, the increase of proinflammatory factors in adipocytes will lead to enhanced transcription of the CYP19 gene encoding aromatase in adipocytes, which in turn will lead to increased expression of aromatase in adipocytes. This article reviews the regulation of male sterility from the angle of the “obesity-inflammation-aromatase” axis.

## 1. Introduction

The World Health Organization predicts that infertility will become the third chronic disease after tumors and cardiovascular and cerebrovascular diseases in the 21st century, with up to 15% of the world's population suffering from infertility, about half of which is caused by male factors [1]. In China, there are nearly 20 million couples suffering from fertility difficulties, among which the infertility caused by the male accounts for about 30%, while in the male infertility patients, the semen quality problem is as high as 90% [2, 3].

There are many causes for male infertility, among which endocrine disorder is an important factor. Up to 70% of infertile men have endocrine dysfunction, such as metabolic syndrome characterized by insulin resistance and obesity. Obesity is the main cause of hypogonadism at present, which associates with impaired gonadal function in males [4]. The fertility of men depends on a certain number and high quality sperm. Spermatogenesis and maturation are a highly complex process involving the fine regulation of sex hormones, testis, Sertoli cells, and epididymal fluid. However, in recent years, it has gradually revealed the fact that obesity affects men's reproductive potential. Too much fat can lead to changes in hormone levels and promote chronic inflammation of the genital tract. High fat content in the scrotal region can also lead to increased scrotal temperature. All of these consequences of obesity destroy the testicular and epididymal microenvironment, which is crucial for sperm production and maturation [5–7]. In recent years, obesity caused by unhealthy lifestyle and social-psychological factors has become a research hotspot. Therefore, clarifying the relationship between obesity and infertility has become the key to research breakthroughs. The purpose of this review is to explore the relationship between the abnormal aromatase axis caused by obesity and male infertility.

## 2. Obese and Inflammation

### 2.1. Related Inflammatory Factors and Chemokines in Obesity

Obesity is a “low-grade chronic inflammatory state” caused by multiple factors [8]. In the state of obesity, lipid deposition leads to the enlargement of adipocyte volume. When adipocyte hypertrophy exceeds its own limit, apoptosis will occur. Necrotic adipocytes secrete various inflammatory factors and chemokines, such as ICAM and VCAM-1. So that the monocyte-macrophages proliferate and migrate to the adipose tissue and have a unique structure, that is, CLSs gather around necrotic fat cells [9]. Adipocytes release more fatty acids and endotoxins, which activate macrophages to transform into M1 macrophages that promote inflammation, and then activate the NF-*κ*B pathway, which significantly increases the release of proinflammatory factors such as TNF-*α*, IL-6, and IL-1*β*, while anti-inflammatory factors are reduced. In addition, hypertrophic adipocytes can also change intracellular signal transduction, increase the expression of proinflammatory cytokines, and ultimately cause chronic inflammation [10].

### 2.2. M1 Macrophage Infiltration under Obesity

The proportion of macrophages in normal adipose tissue to total cells is only 10%, while in obese tissues, this proportion can be as high as 50%, and the increase is mainly M1 macrophages that secrete proinflammatory factors [11]. The accumulation and infiltration of ATMs cause aseptic inflammation and break the balance between M1 macrophages and M2 macrophages, resulting in the polarization of M2 macrophages secreting anti-inflammatory factors towards M1 macrophages secreting proinflammatory factors [12]. Then, M1 macrophages will secrete a large amount of proinflammatory factors. This causes the obese people to gradually change from a “low-grade chronic inflammation state” locally to the whole body, thus forming a vicious circle [13, 14].

### 2.3. Tissue Hypoxia and Chronic Inflammation Caused by Obesity

Local hypoxia in adipose tissue is also one of the mechanisms of chronic inflammation, and this local hypoxia is caused by insufficient blood flow supply due to the rapid growth of adipose tissue [15]. In the case of low oxygen content, the cell itself has a defense mechanism to adapt to this hypoxic environment. This cell's defense response to hypoxia is achieved through the activation of specific transcription factors, including: HIF-1*α*, GLUT1, HO-1, PDK1, and VEGF, which increase the expression of factors produced by local hypoxia. It further recruits macrophages to infiltrate adipose tissue, leaving the body in a state of low-grade inflammatory response [16].

### 2.4. Oxidative Stress in Obesity

In the case of a high-carbohydrate or high-fat diet, excessive glucose and fatty acids generate pyruvate, CoA, and other reducing metabolites. Then, the above-mentioned substrates enter the mitochondria for oxidation, which enhances the activity of the mitochondrial respiratory chain and increases single electron transfer and ROS production [17]. Due to the chronic low-level inflammatory response in obese patients, inflammation activates a variety of immune cells to produce a large number of free radicals, aggravating oxidative stress. Oxidative stress further aggravates cell oxidative damage and accelerates cell senescence. Senescent adipocytes recruit macrophages, release a variety of proinflammatory cytokines, and promote inflammation [18].

## 3. Body Inflammation and Expression of Aromatase

### 3.1. The Biological Activity of Aromatase

**Figure 1:**
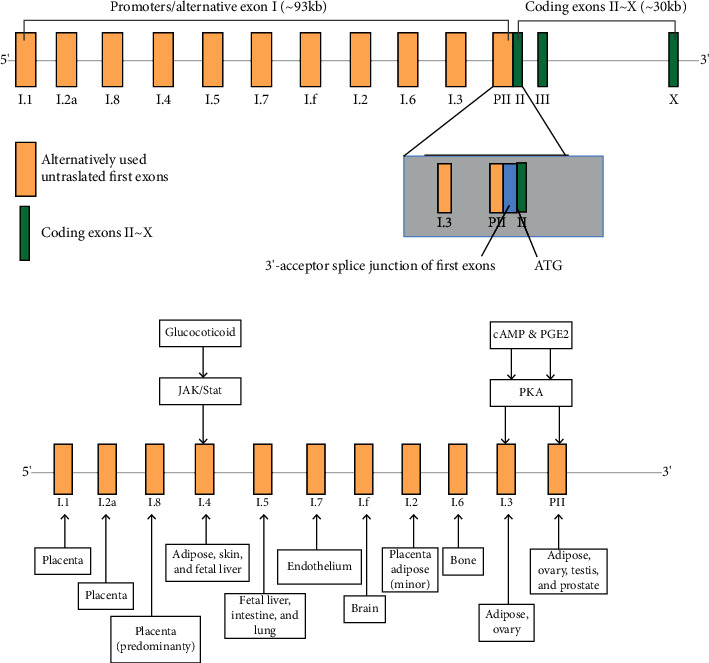
Encoding CYP19A1 gene structure of aromatase.

Aromatase belongs to the cytochrome P450 superfamily, which is encoded by the CYP19A1 gene located on chromosome 15 (15q21). The entire CYP19 gene is 123 kb, of which about 30 kb containing 9 exons (II-X) that encode the aromatase protein and 93 kb containing untranslated first exons that are controlled in a tissue-specific manner. There are a variety of tissue-specific promoters in the 5′-end nontranslational region, including PI.1, PI.2a, PI.8, PI.4, PI.5, PI.7, PI.f, PI.2, PI.6, PI.3, and PII [19]. The promoter selectively splices the nontranslational area (5-UTR) of its own exons I 5′ side onto the splice site (AG/GACT) at the 38 kb upstream of the translation initiation site (ATG) on exon II (Figure 1) [20, 21]. The regulatory factors of CYP19 gene include cis-acting factors such as CRE together with ER, and trans-acting factors such as growth factor together with TBP.

Aromatase is composed of two proteins, NADPH cytochrome P450 and specific cell P450 aromatase. The former provides reductive equivalent NADPH+H^+^ and electron transfer for hydroxylation catalyzed by P450arom, while the latter provides steroid hormone binding sites for catalytic synthesis of estrogen [22]. Aromatase is a key enzyme in the synthesis of estrogen, which can catalyze the irreversible conversion of testosterone and androstenedione into estrogen, which plays an important role in the life activities of normal individuals. Due to alternative use of multiple promoters, the transcription level of CYP19 and the activity of aromatase in different tissues are different. In humans, aromatase is expressed in many cells and tissues, including Leydig cells, Sertoli cells, granulocytes, luteal cells, placental cells, neurons, fibroblasts, vascular smooth muscle cells, preadipocytes, chondrocytes, and osteoblasts, etc. [23, 24].

A natural mutation in the male aromatase gene may lead to testicular abnormalities, endocrine disorders, and altered sperm parameters. Increased aromatase activity can lead to decreased testosterone levels, which is one of the mechanisms leading to male hypogonadism. AI can inhibit aromatase activity, correct the condition of “low androgen and high estrogens,” and improve the spermatogenic function of testis. More and more attention has been paid to the use of AI in the treatment of spermatogenesis dysfunction. AI is especially suitable for patients with spermatogenesis dysfunction with low androgen level and high estrogen level (quantified by T/E_2_ ratio, which is currently considered as low limit of 10) [25]. Although clinicians have attached increased importance to the therapeutic effect of AI on spermatogenic disorders, more in-depth studies are needed in terms of their applicable population and mechanism of action.

### 3.2. Inflammatory State Induces the Expression of Aromatase

**Figure 2:**
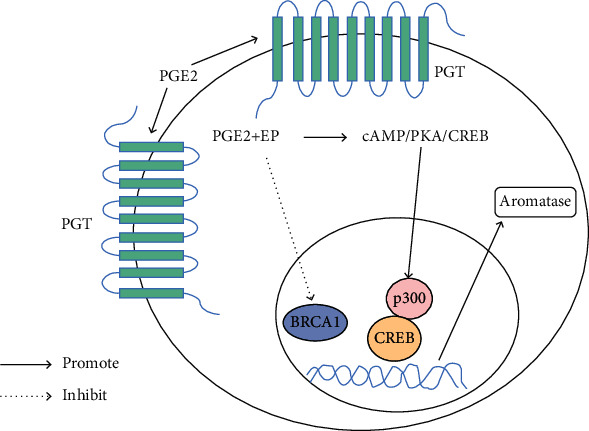
Signal transduction pathway of aromatase expression regulated by PGE2 through PGT. PGT is a trans-membrane domain transporter that carries extracellular PGE2 into cytoplasm, which activates the CAMP/PKA/CREB pathway by binding to its receptors EP2 and EP4, thus resulting in enhanced interaction between CREB, p300, and aromatase promoter I.3/II. PGE2 also decreased the amounts of BRCA1 and inhibited the interaction of BRCA1 and aromatase promoter I.3/II; then, CYP19 transcription and aromatase activity are enhanced ultimately.

Obesity is now accepted as a low-grade, chronic, and systemic inflammatory disease, which is predominantly characterized by an increase of M1 macrophages and the inflammatory mediators in adipose tissue [26, 27]. And the obesity-associated adipose tissue inflammation leads to increased CYP19A1 expression in males, which leads to excessive aromatase in obese men. The CYP19 gene contains several cis-regulatory elements [28, 29], of which the CRE is the major regulator. The cAMP activates the downstream cAMP-dependent protein kinase that is also called PKA and causes CREB phosphorylation, thereby coupling CRE latterly. The formation of the cAMP/PKA/CREB signaling pathway leads to subsequent signal transduction and upregulation of aromatase expression. Discovery showed that insulin concentration and sensitivity could interfere with various signal pathways, which were mainly based on the cAMP-dependent signaling pathway, thus affecting the expression of CYP19. The high expression of proinflammatory mediators such as PGE2, TNF-*α*, and IL-6 in M1 macrophages can induce the expression of aromatase in preadipocytes by amplifying the inflammatory effect. And the significant regulatory effect of PGE2 on CYP19 is worthy of attention. The PGT removes PGE2 from the extracellular milieu and delivers it to the cytoplasm. Thereby, PGE2 activates the CAMP/PKA/CREB pathway through binding to EP2 and EP4, thus resulting in enhanced interaction between CREB, p300, and aromatase promoter I.3/II [30]. Moreover, PGE2 decreases the amounts of BRCA1, a repressor of aromatase transcription, which reduced interaction between BRCA1 and the aromatase promoter I.3/II. In conclusion, CYP19 transcription and aromatase activity are enhanced ultimately (Figure 2).

Meanwhile, the results of Kotha Subbaramaiah showed that M1 macrophages infiltrated in adipose and breast tissues of obese mice, with increased content of inflammatory mediators and aromatase. These results suggest that the expression of the CYP19 gene may be influenced by proinflammatory mediators, which may in turn change the aromatase content and activity in obese mice. Collectively, these findings suggest that obesity-induced inflammation interfering with the cAMP-dominated signal transduction pathway through secondary IR and related inflammatory factors, stimulating CYP19 transcription and elevating the aromatase level.

In recent years, the biological effects of EGCG and TGR5 on improving obesity have attracted much attention. It has been found that EGCG administration can improve the interference of nutritional obesity on insulin signal, reduce the accumulation of lipids in liver tissue, and interfere with the TLR4-mediated inflammatory response pathway and the key molecule of insulin signal pathway in liver tissue, thus balancing the redox state, relieving inflammation [31–33]. Additionally, EGCG can also make the composition of its intestinal microbiota significantly change and LPS into the blood reduce, thus reducing endotoxemia, alleviating IR and improving low-grade, chronic obesity-associated inflammation. The findings of Hassan et al. showed that EGCG could improve the male infertility with the EGCG administration, suggesting that EGCG could improve the sterility of male rats by ameliorating inflammation, thereby affecting the expression of aromatase [34]. Furthermore, TGR5 has been found to have anti-inflammatory effects by inhibiting NF-*κ*B, thus inhibiting proinflammatory cytokine production. The mice with the TGR5 gene knocked out showed severe hyperlipidemia, steatosis, IR, and inflammation.

PPARs are members of the nuclear receptor superfamily and have three subtypes, PPAR*α*, PPAR*β*/*δ*, and PPAR*γ*, which have the properties of anti-inflammation, anti-TLR4 inflammatory response pathway, and anti-NF-*κ*B [35, 36]. PPARs are found in many tissues, such as the liver, heart, skeletal muscle, and brown adipose tissue. The localization of PPARs in mouse testis by Gang Wang et al. showed that PPAR*α* was mainly located in the nucleus of Leydig cells and PPAR*β*/*δ* mainly in Sertoli cells, while PPAR*γ* mainly distributed in the nucleus of the sperm cell. It has been found that the activation of PPARs may regulate the expression of aromatase directly or indirectly by regulating the transcription of NF-*κ*B, ROS enzyme-relevant genes, and RNA metabolism together with anti-inflammatory and antioxidative pathways [37].

## 4. Aromatase Expression Disorder and Male Infertility

### 4.1. Aromatase Overexpression Leads to Hormone Disorder

**Figure 3:**
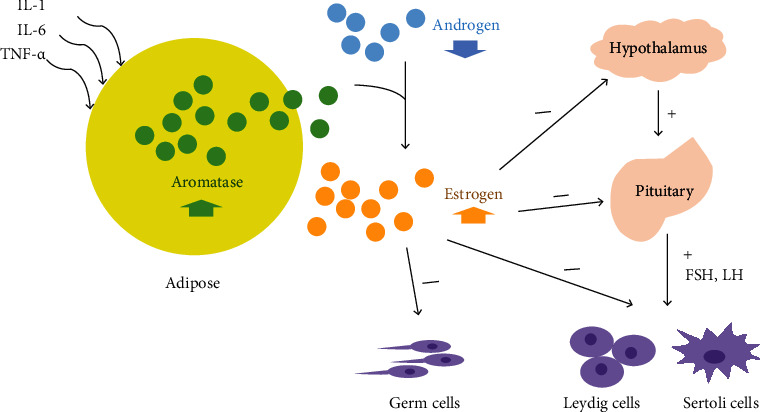
Effect of aromatase axis disorder on spermatogenic system. During inflammation, the secretion of proinflammatory factors such as IL-1, IL-6, and TNF-*α* increases the expression of aromatase in adipose tissue. The high expression of aromatase makes androgens irreversibly converted into estrogens, breaking the balance between male and female hormones. That is, the body androgen level drops and the estrogen level rises. Therefore, on the one hand, it can directly regulate the testicular stromal cells so that spermatogenesis cannot proceed normally. On the other hand, the HPG axis is inhibited by inhibiting the release of FSH and LH as well as Leydig and Sertoli cells, thus hindering the process of spermatogenesis.

Aromatase, as a rate-limiting enzyme for the irreversible conversion of androgens to estrogens, leads to the overconversion of androgens to estrogen in men, resulting in a high concentration of estrogen and low concentration of androgens, when its activity increases. Although estrogen has always been regarded as a typical female hormone, more and more studies show that estrogen not only maintains a certain amount in the male body but also plays an important role in male reproduction in recent years. Therefore, the balance between estrogen and androgen is an important part of male fertility, and the aromatase expression will be particularly important in this balance. Excessive estrogen inhibits the release of FSH and LH by inhibiting the HPG axis, thereby further reducing androgen levels. Spermatogenesis proceeds with the rigorous regulation of the above hormones. So when the balance of these hormones is disturbed, it will bring a negative effect on spermatogenesis. Therefore, the high expression of aromatase can disrupt the hormone balance in the male body, hinder the process of spermatogenesis, and lead to male infertility. In recent years, AI administration has received extensive attention in the field of male infertility. It can correct the condition of “low androgen level and high estrogen level” and then improve the spermatogenic function of the testis. It also relieves estrogen's negative feedback inhibition of the HPG axis, which in turn promotes the release of FSH and LH from the pituitary gland (Figure 3) [25].

### 4.2. The Receptor Sensitivity of Estrogen Is Enhanced in the State of High Expression

Studies have shown that when estrogen levels in male rats are elevated, the expression of estrogen receptors ER*α* and ER*β* in sperm cells is increased. This suggests that with increased levels of estrogen, its sensitivity is also increased, which is also detrimental to spermatogenesis [38].

### 4.3. Damage of Leydig Cells Induced by Overexpression of Aromatase

There are two types of macrophages in the testis. One is macrophage ED1^+^ that moves from the peripheral blood to testicular tissue. This type of macrophage increases during chronic inflammation of the testis. The other is macrophage ED2^+^, which is testis tissue-resident. It is less active and produces lower levels of inflammatory factors than the peritoneal macrophages. Under physiological conditions, the macrophages in testis can express IL-1, IL-6, TNF-*α*, and other major inflammatory factors, regulating the function of the testis.

In the male reproductive system, Leydig cells are important sites for aromatase to realize its biological activity. In animal experiments and clinical patients, we can find that when aromatase is overexpressed, excess estrogen causes macrophage activation in Leydig cells, which in turn are devoured by adjacent macrophages. This results in damage to the blood-testosterone barrier in the end. Apparently, it will adversely affect spermatogenesis and storage, thereby impairing male fertility [39, 40].

**Figure 4:**
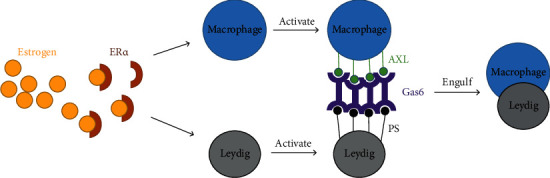
Mechanism of the estrogen-induced phagocytosis of Leydig cells by macrophages. Estrogen acts on macrophages and Leydig cells through estrogen receptor ER. In Leydig cells, estrogen activates its cell surface overexpression of phospholipid amino acid, PS, and growth specificity gene 6, Gas6. Through the Gas6, the PS molecules bind to AXL, one of the TAM receptor tyrosine kinase subfamily, which are overexpressed by macrophages after the estrogen stimulation. Namely, the PS molecules are an “eat me” signal to attract the macrophage cell to engulf the Leydig cells.

In addition, studies have shown an increase in the number of macrophages in azoospermia patients. Overexpression of aromatase and high levels of estrogen in testis activate the testis macrophages. At the same time, the Leydig cells are also activated and release specific molecules, making macrophages through the AXL-Gas6-PS pathway engulf the Leydig cells. Again, it will damage the blood-testosterone barrier and induce male sterility. The mechanism of the estrogen-induced “eat me” signaling in Leydig cells is illustrated below (Figure 4).

## 5. Prospects

In conclusion, the obesity-inflammation-aromatase axis severely impairs male fertility and causes male infertility. Although some studies have found that the intervention of EGCG and TGR5 can improve the body's inflammatory state to a certain extent, thereby improving infertility, we should realize that obesity not only leads to male infertility from this aspect of the “obesity-inflammation-aromatase axis” but also causes male infertility from obesity-induced leptin resistance and oxidative stress damage, etc. [4, 41]. In addition, we should be aware that male infertility is not caused solely by obesity and, where necessary, consider obesity as a common or synergistic factor in infertility, as well as other etiological factors.

## Abbreviations

AI: Aromatase inhibitor
ATMs: Adipose tissue macrophage
cAMP: Cyclic adenosine monophosphate
CLSs: Crown-like structures
CoA: Coenzyme A
CRE: Cyclic adenosine monophosphate- (cAMP-) response element
CREB: CRE binding protein
CYP: Cytochrome P450 proteins
EGCG: Epigallocatechin-3-gallate
ER: Estrogen receptor
GLUT1: Glucose transporter-1
HIF-1*α*: Hypoxia inducible factor-1*α*
HO-1: Heme oxygenase-1
IR: Insulin resistance
ICAM: Intercellular adhesion molecule
LPS: Lipopolysaccharide
PDK1: Pyruvate dehydrogenase kinase-1
PGT: Prostaglandin transporter
PKA: Protein kinase A
PPAR*γ*: Peroxisome proliferator-activated receptor *γ*
ROS: Reactive oxygen species
TBP: TATA binding protein
TGR5: G protein coupled bile acid receptor 5
TLR4: Toll-like receptor 4
VCAM-1: Vascular cell adhesion molecule-1
VEGF: Vascular endothelial growth factor.
